# Presentation of AIDS with Disseminated Cutaneous and Visceral Leishmaniasis in Iran

**DOI:** 10.1155/2015/563851

**Published:** 2015-05-05

**Authors:** Mohammadali Davarpanah, Masumeh Rassaei, Fatemeh Sari aslani

**Affiliations:** ^1^Infectious Disease, Shiraz HIV/AIDS Research Centre, Shiraz University of Medical Sciences, Shiraz, Iran; ^2^Shiraz University of Medical Sciences, Shiraz, Iran; ^3^Pathology Department, Shiraz University of Medical Sciences, Shiraz, Iran

## Abstract

Leishmaniasis is an infectious disease in form of visceral (VL), cutaneous (CL), and mucocutaneous (MCL) leishmaniasis. Immunocompromised patients have increased risk of *Leishmania* infection, especially in endemic areas for visceral leishmaniasis, where in the world HIV/VL coinfection has become endemic. The case here suffers from both AIDS and visceral-cutaneous leishmaniasis. We report an Iranian woman with disseminated cutaneous and visceral leishmaniasis who became positive for HIV test.

## 1. Introduction

Leishmaniasis is a vector-borne infectious disease in form of visceral (VL), cutaneous (CL), and mucocutaneous (MCL) leishmaniasis. About 90% of cutaneous leishmaniasis occurs in Iran, Saudi Arabia, Syria, the Middle East, Afghanistan, and Brazil [1].

There are probably much more cases of VL than those registered and have been estimated in the European countries [2], as pointed out by Lachaud et al. for France [3].

Visceral leishmaniasis (VL) usually occurs in the same areas endemic for CL.

Visceral leishmaniasis is endemic in several parts of our country [4]. VL is also endemic in nine European countries. The World Health Organization has estimated a total of VL incidences of approximately 410–620 cases each year during 2003 to 2008 in these European countries [2]. Different types of* Leishmania* are considered as major health problems in the world.* L. tropica* and* L. major*, which are the causative agents of cutaneous leishmaniasis, have been reported in the Middle East. Visceral leishmaniasis (VL) is caused by* L. donovani* complex including* L. donovani* in Asia and* L. infantum* in the Middle East. Visceral leishmaniasis presents with fever, weight loss, hepatomegaly, splenomegaly, and pancytopenia [5].

Among immunosuppressed cases due to acquired immune deficiency syndrome (AIDS), malnutrition, and neoplasms along with immunosuppressive therapy, the risk of visceral progression is accentuated. In southern European area, visceral leishmaniasis has become endemic among HIV infected patients [1]. In areas where VL is endemic, HIV virus increases the risk of VL up to 100–1000-fold. In AIDS patients, VL is more likely to become chronic. Also, common clinical manifestations of VL such as fever, weight loss, and hepatosplenomegaly do not always occur among coinfected cases [5]. The current case is an HIV positive patient who presented with diffuse cutaneous and visceral leishmaniasis as initial presentation of AIDS.

## 2. Case Report

**Figure 1:**
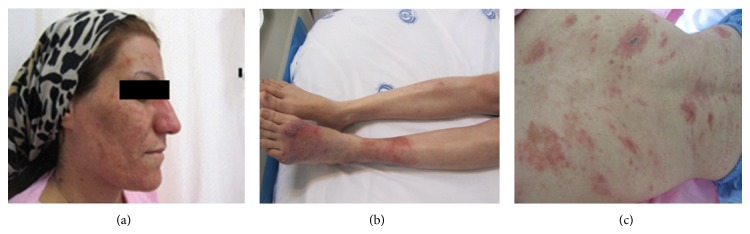
The patient's skin lesions (a) on face, (b) on lower extremity, and (c) on back.

Our case is a 36-year-old woman, married, resident in Marvdasht (a small town in Fars province) who presented with diffuse erythematous indurated plaque-like lesions distributed on face, trunk, abdomen, back, and upper and lower extremities (Figure 1) for 6 months prior to admission and received some topical treatment without any improvement. In physical examination no evidence of lymphadenopathy, hepatosplenomegaly, or fever was seen. In the laboratory data, liver and renal function tests, ANA, and ESR value were normal. Hematocrit and platelet count were within normal limits but she showed severe leukopenia (leukocyte count 530 per milliliter).

**Figure 2:**
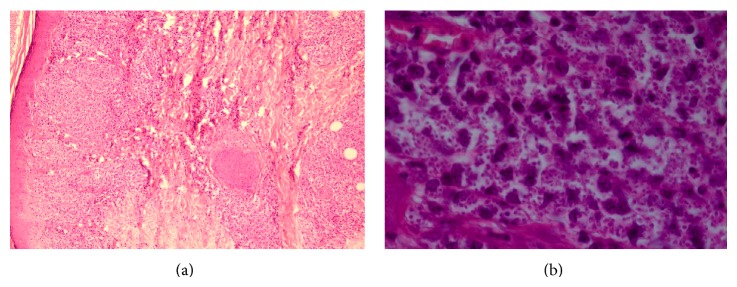
Skin biopsy (a). A dense mainly histiocytic infiltrate in the papillary and reticular dermis (H&E x100) (b). Many histiocytes loaded by Leishman bodies (H&E x400).

**Figure 3:**
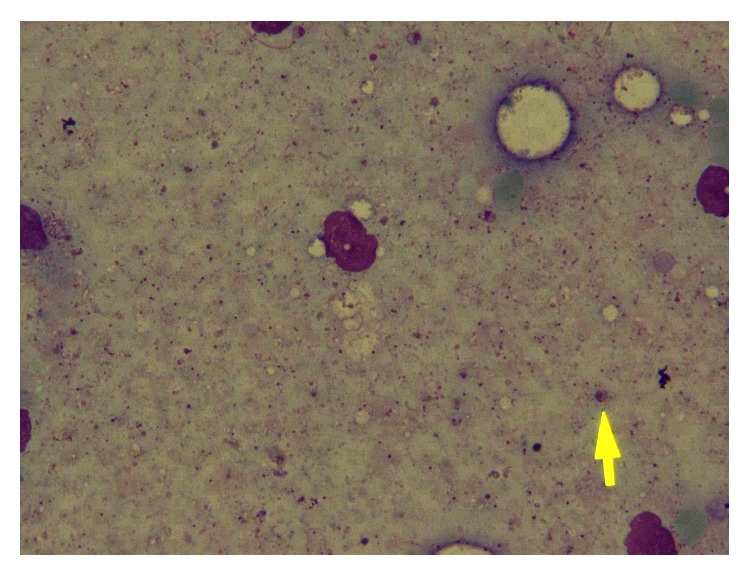
Bone marrow smear revealed a few Leishman bodies, one of them showed by arrowhead (Wright's stain x1000).

Skin biopsy was performed for her and Leishman bodies were reported by pathologist (Figure 2). Due to severe leukopenia, bone marrow biopsy was performed which revealed normocellular marrow and few Leishman bodies (Figure 3). To determine the species of* Leishmania*, we used PCR and* L. major* was confirmed.

Conventional amphotericin B was prescribed for her. Skin lesions were partially improved after about ten days of treatment, but leukopenia was persistent. To find the reason of leukopenia, HIV antibody was checked by ELISA method. Then positive result was confirmed by the western blot test. Initial CD4+ T cell count was 120/microliter.

The patient was treated with antiretroviral drugs regimen including lamivudine, zidovudine, and efavirenz. After prescription of antiretroviral therapy, her leukocyte count was partially increased and the patient was discharged after four weeks of amphotericin B treatment with leukocytes count 2700/microliter. In the outpatient's follow-up two weeks later, her skin lesions became better in appearance than in previous visit and in the secondary bone marrow biopsy, Leishman bodies disappeared.

## 3. Discussion

Cutaneous leishmaniasis manifests with chronic skin lesions. Visceral leishmaniasis presents with hepatosplenomegaly, fever, weight loss, and pancytopenia. Disseminated cutaneous leishmaniasis exists as a syndrome with 100 acne papules in immunocompetent patients in Brazil. It can be seen in a few patients of AIDS and organs transplant recipients [1]. In Iran* L. infantum* has been isolated from cutaneous type [6]. In Iran and Fars province,* L. infantum *is the causative agent of VL [6, 7]. But* L. tropica* has also been rarely reported as the causative agent of VL in Iran [1, 8]. In another study,* L. major* and* L. tropica* have been reported in VL patients in Iran [7].

Immunosuppression increases the risk of visceral involvement by* Leishmania*. Visceral leishmaniasis has become an important opportunistic health problem in Southern Europe. In other regions of the world the disease can be seen in transplant recipients and cellular immune deficient patients. Another problem in immunodeficient individuals is relapse of VL that can be seen, even several years after treatment. In late stage of HIV infection, VL may occur as an opportunistic infection [1]. Coinfection of VL and HIV has been reported in 1700 cases in 33 countries of the world. India has the highest number of cases [9]. In CD4+ T cell count >50/microliter, all typical clinical symptoms of VL including hepatosplenomegaly, weight loss, and pancytopenia can be seen but in CD4+ T cell count <50/*µ*L the disease appears with atypical clinical manifestations. It can present with lung, gastrointestinal tract, and pleural involvement and aplastic anemia [1].

Prevalence of opportunistic leishmaniasis has fallen with new antiretroviral drugs but in endemic areas of the world coinfection of HIV/VL is rising [1]. Visceral leishmaniasis is an opportunistic disease in HIV patients in endemic areas of Iran [10]. Disseminated cutaneous leishmaniasis has been reported in a small number of patients with AIDS and organ transplant recipients [1]. Two cases of disseminated cutaneous leishmaniasis have been reported in Iran in 2010 among HIV negative individuals [11].

In Jahrom (a small town in Fars province) in 2009 Pourahmad et al. reported a case of HIV/visceral-cutaneous leishmaniasis coinfection that presented with lymphadenopathy, skin lesions 6 months after HIV diagnosis with CD4+ T cell count 195/microliter. In the skin biopsy, bone marrow aspiration, and lymph node biopsy specimens, Leishman bodies were reported [9]. In 2010, Jafari et al. have reported two cases of concurrent HIV and visceral-cutaneous leishmaniasis. The first case was HCV/HIV coinfection with CD4+ T cell count 180/microliter and leproid nodules on the face and ears. The second was HIV positive with CD4+ T cell count 180/microliter and lymphadenopathy in the neck and axilla and mild splenomegaly. In both patients' biopsies of bone marrow and skin, Leishman bodies were found. In both patients,* Leishmania* were of* L. tropica* type [8].

In Khorasan province of Iran, Shafiei et al. followed up 500 asymptomatic HIV positive patients for leishmaniasis. They were screened by direct agglutination test (DAT) where 49 out of them had positive DAT and 9 seropositive individuals had antibodies against* Leishmania infantum*. Shafiei declared that, based on the findings of the study, visceral leishmaniasis in endemic areas of Iran has become an opportunistic infection in HIV positive patients [10]. Our case is a patient with disseminated cutaneous and visceral leishmaniasis who became positive for HIV antibody test. All three previous reported cases of HIV/VCL coinfection in Iran were HIV positive where leishmaniasis occurred as opportunistic infection in them. In our case unlike previous cases, visceral and disseminated cutaneous leishmaniasis was initial presentation of AIDS. The subtype of* Leishmania* among HIV/VL coinfected patients in Iran has been* L. tropica* while* L. major* was isolated from skin and bone marrow tissues of our case.
